# In vitro model of brain endothelial cell barrier reveals alterations induced by *Plasmodium* blood stage factors

**DOI:** 10.1007/s00436-023-07782-x

**Published:** 2023-01-25

**Authors:** Teresa F. Pais, Carlos Penha-Gonçalves

**Affiliations:** Instituto Gulbenkian de Ciência, Rua da Quinta Grande, 6, 2780-156 Oeiras, Portugal

**Keywords:** Cerebral malaria, Brain endothelium, BBB, *Plasmodium*

## Abstract

Cerebral malaria (CM) is a severe neurological condition caused by *Plasmodium falciparum*. Disruption of the brain-blood barrier (BBB) is a key pathological event leading to brain edema and vascular leakage in both humans and in the mouse model of CM. Interactions of brain endothelial cells with infected red blood cells (iRBCs) and with circulating inflammatory mediators and immune cells contribute to BBB dysfunction in CM. Adjunctive therapies for CM aim at preserving the BBB to prevent neurologic deficits. Experimental animal and cellular models are essential to develop new therapeutic strategies. However, in mice, the disease develops rapidly, which offers a very narrow time window for testing the therapeutic potential of drugs acting in the BBB. Here, we establish a brain endothelial cell barrier whose disturbance can be monitored by several parameters. Using this system, we found that incubation with iRBCs and with extracellular particles (EPs) released by iRBCs changes endothelial cell morphology, decreases the tight junction protein zonula occludens-1 (ZO-1), increases the gene expression of the intercellular adhesion molecule 1 (ICAM-1), and induces a significant reduction in transendothelial electrical resistance (TEER) with increased permeability. We propose this in vitro experimental setup as a straightforward tool to investigate molecular interactions and pathways causing endothelial barrier dysfunction and to test compounds that may target BBB and be effective against CM. A pre-selection of the effective compounds that strengthen the resistance of the brain endothelial cell barrier to *Plasmodium*-induced blood factors in vitro may increase the likelihood of their efficacy in preclinical disease mouse models of CM and in subsequent clinical trials with patients.

## Introduction

Cerebral malaria (CM) is the most lethal form of malaria in *Plasmodium falciparum* (*Pf*)-infected children under 5 years, with case fatality rates that can reach 15% (Seydel et al. 2015). Moreover, 25% of children who survive CM suffer motor and/or behavior impairments and long-term cognitive and hearing deficits due to disease-induced cerebral lesions (reviewed in Idro et al. (2010)).

Sequestration of iRBCs in the brain microvasculature is a hallmark of CM pathology observed in post-mortem tissue (Pongponratn et al. 2003; Taylor et al. 2004). Malaria clinical symptoms occur during the intraerythrocytic multiplication of the parasite, which alters the function and structure of iRBCs promoting binding to endothelial cells (reviewed in De Koning-Ward et al. (2016)). This phenomenon, along with elevated serum levels of TNF, contributes to an increase in adhesion molecules (intercellular adhesion molecule-1 (ICAM-1), vascular cell adhesion molecule 1 (VCAM-1), and E-selectin (Turner et al. 1994; Dunst et al. 2017)) and to a decrease in endothelial tight junction proteins (ZO-1 and occludin) (Brown et al. 1999). Interaction of iRBCs with endothelial cells also leads to activation of the coagulation cascade (reviewed in Francischetti (2008)) and vessel obstruction, which affects brain blood perfusion (Warrell et al. 1988). Altogether, these mechanisms compromise the BBB integrity causing vascular leakage with brain edema and intracranial hypertension (Newton et al. 1997; Seydel et al. 2015; Mohanty et al. 2017). In children with CM, a magnetic resonance imaging (MRI) study associates brain swelling with compression of respiratory centers and a poor outcome due to respiratory failure (Seydel et al. 2015).

Reproducing to a considerable extent the human cerebral malaria pathology, the mouse model—C57BL/6 mice infected with *Plasmodium berghei* ANKA (*Pba*)-infected RBCs—develops neurologic symptoms such as ataxia and hemiplegia that rapidly progress to coma and death associated with brain vascular damage, loss of BBB integrity, and brain edema (van der Heyde et al. 2001; Ball et al. 2013; Nacer et al. 2014; Hoffmann et al. 2017). We recently found that the brain endothelium also senses the blood infection through STING1 activation and induction of CXCL10, a chemokine involved in the recruitment of CD8^+^ T cells to the brain (Pais et al. 2022). Accumulation of iRBCs in the brain and parasite-specific CD8^+^ T cells that interact with the brain endothelium through cross-presentation of parasite antigens (Howland et al. 2013) are essential for disruption of the BBB and development of experimental CM (Baptista et al. 2010).

The BBB is a selective semipermeable structure that plays a key role in protecting the brain from noxious blood alterations that can compromise neuronal function. It is composed by microvascular endothelial cells surrounded by pericytes and reinforced by astrocytes end-feet. The tight junction localized on the apical side of the endothelium forms a barrier that restricts paracellular diffusion (reviewed in Abbott et al. (2006)).

Several studies assessed the mechanisms of BBB disruption in CM by analyzing interactions of iRBCs (reviewed in Polimeni and Prato (2014)), iRBC-derived extracellular vesicles (Mantel et al. 2016; Pais et al. 2022), ruptured iRBCs (Gallego-Delgado et al. 2016), and *Plasmodium*-derived products (Pal et al. 2016) with brain endothelial cells in vitro. Specific domains of the *Pf* erythrocyte membrane protein 1 (PfEMP1) family, encoded by *var* genes, were shown to interact with receptors on the endothelial cell membrane such as CD36 (Baruch et al. 1996), ICAM-1, and the endothelial protein C receptor (EPCR) (Turner et al. 2013; Lennartz et al. 2017; Adams et al. 2021). Moreover, it has been shown that ICAM-1-dependent uptake of whole *Pf*-infected RBCs may also contribute to endothelial cell damage (Adams et al. 2021). Other studies, mostly in cell lines and in non-brain-specific endothelial cells, found that the endothelial-iRBC interaction increases expression of adhesion molecules, decreases the levels of tight junction proteins and transendothelial resistance (TEER), or increases BBB permeability to low molecular solutes (reviewed in Polimeni and Prato (2014)). Nevertheless, a single-cell model and systematic way to assess the several parameters associated with BBB dysfunction is lacking in cerebral malaria research.

We established an in vitro endothelial cell barrier model using primary endothelial cells obtained from mouse brain vessels. We show that this experimental system is suitable to study the effect of *Pba* blood stages on endothelial cell barrier properties and adhesion molecule gene expression. With this system, we found that not only iRBCs but also EPs isolated from the iRBC cell culture supernatant reduce TEER, ZO-1, and Claudin 5 levels and increase the permeability to a low molecular solute. Importantly, to address the role of specific molecules in BBB disruption, primary cultures may be obtained from available mice deficient for several signaling pathways. Moreover, potential compounds that protect the BBB disruption can be tested in this system providing insights into new adjunctive therapies in addition to chemotherapy, which may improve the clinical outcome of CM.

## Material and methods

### Ethics statement

All procedures involving laboratory mice were in accordance with national (Portaria 1005/92) and European regulations (European Directive 86/609/CEE) on animal experimentation and were approved by the Instituto Gulbenkian de Ciência (IGC) Ethics Committee and the Direcção-Geral de Alimentação e Veterinária, the national authority for animal welfare.

### Isolation of brain vessels and culture in inserts

C57BL/6 mice with 6–8 weeks of age were used to obtain the brain vessels. Immediately after euthanasia, mice were exsanguinated by cardiac puncture. The brains were removed from the skull and kept in a Petri dish with HBSS medium (Biowest) on ice. Then, the brains were transferred to a freezer block covered with gaze and the meninges removed by rolling a cotton swab over them. Brain vessels were then isolated adapting a previously described procedure (Howland et al. 2015). Briefly, brains were minced with a blade in 1 ml of HBSS medium and aspirated with a syringe. The tissue was then homogenized pushing it five times through a 23G needle. This lysate was mixed with an equal volume of cold 30% (wt/vol) dextran (MW ~60 kDa) (Sigma) solution and centrifuged at 10,000g for 15 min. After centrifugation, the myelin layer in the top was removed and the pellet containing the vessels was suspended in DMEM and passed through a 40-μm cell strainer followed by 5 ml of medium to wash away other cells and myelin debris. The strainer was back-flushed with 5 ml of DMEM into a 60-mm Petri dish to collect the brain vessels of 2–3 brains supplemented with 1mg/ml of collagenase type IV (Millipore), 10 μg/ml DNAse (Roche), and 2% FBS and followed by incubation for 2–3 h at 36.5 °C in an orbital shaker. The digested vessels were then centrifuged and suspended in EGM™-2 Endothelial Cell Growth Medium-2 BulletKit™ (LONZA) containing 4 μg/ml of puromycin and plated on hanging cell culture inserts with polyethylene terephthalate (PET) filters with a pore size of 5 μm (cat. nr. MCMP24H48, Millipore) at a ratio of one brain for 2.5 inserts. The inserts were previously coated with ECM Gel from Engelbreth-Holm-Swarm murine sarcoma (matrigel matrix) (SIGMA) for 2 h in 24-well plates at 37 °C (37°C, 5% CO2). Every 4 days, the medium in the top and bottom of the insert (0.5 ml in each compartment) was changed for fresh medium.

### Measurement of transendothelial electrical resistance (TEER)

The 24-well plates containing the cells cultured on inserts were removed from the tissue culture incubator (37°C, 5% CO2) and allowed to equilibrate to room temperature. TEER values were measured before changing the medium using an EVOM2 voltmeter with STX-2 electrodes (Millipore). To calculate TEER (Ω (Ohms) × cm^2^) in each insert culture, electrical resistance across a matrigel-coated insert without cells was subtracted from the readings obtained on inserts with cells and then multiplied by the surface area of the insert (0.3 cm^2^).

### Preparation of synchronized iRBCs and extracellular particles (EPs)

C57BL/6 mice with 8 weeks of age were intraperitoneally injected with 10^6^ *Plasmodium berghei* ANKA-GFP (PbA)-iRBCs. Parasitemia was determined as the percentage of GFP^+^ RBCs, using flow cytometry analysis (FACScan Cell Analyzer; Becton Dickinson). At day 5–6 post-infection when parasitemia was 10–20%, blood was collected (between 13:00h and 15:00h) by cardiac puncture with a syringe containing 100 μl of heparin. The collected blood was suspended in 5 ml of RPMI1640 medium and centrifuged at 500g for 10 min. iRBCs were then synchronized in RPMI1640 medium supplemented with FBS, to a final concentration of 20%, and neomycin (100 μg/ml) in 250-ml culture flasks flushed with a gas mixture of 5% CO2, 5% O2, and 90% N2 using a 0.2-μm filter unit connected to the gas hose as previously described (Janse et al. 2006). After 16 h, RBCs and iRBCs were collected by centrifugation and the supernatant used to collect EPs. The pellet was resuspended in PBS containing 2% BSA and mature-stage iRBC were selected and concentrated using automatic magnetically activated cell sorting (MACS) as previously described (de Moraes et al. 2016). Supernatant from RBCs and iRBCs was further centrifuged at 1600g for 20 min to remove lysed cells. The resultant supernatant was further centrifuged at 20,000g for 20 min to obtain a pellet with EPs. Medium and PBS solutions used to prepare EPs were previously filtered through a 0.2-μm filter.

### EP flow cytometry analysis

EPs, RBCs, and iRBCs were suspended in 0. 2-μm filtered PBS and analyzed by flow cytometry (Fortessa, BD) using side scatter from the blue-violet laser (SSC-BV) to detect smaller particles. PBS was used to set the background cytometric signal.

EPs were also stained with phycoerythrin (PE)-labeled anti-mouse TER119 antibody (Biolegend) and PerCP/Cy5.5-labeled Annexin V (Biolegend) in calcium binding buffer (0.01 M HEPES, pH 7.4; 0.14 M NaCl; 2.5 mM CaCl 2). After staining, the preparation was washed in PBS by centrifuging at 20,000g.

### Monolayer permeability to Lucifer yellow (LY)

After 22–24 h of endothelial cell culture with the different stimuli, the inserts were removed to a new 24-well plate containing 0.5 ml of HBSS medium without phenol red and with 10 mM HEPES and 2% of FBS (transport buffer) per well. LY (Sigma-Aldrich) at 100 μM was added in 0.5 ml of transport buffer to the insert and incubated at 37 °C for 1 and 2 h. At these time points, 100 μl was collected from the lower chamber and the amount of LY detected by optical absorbance in a plate reader (Glomax Explorer) (excitation: 405 nm and emission: 500–550 nm).

### Immunofluorescence staining

Inserts with cell cultures were fixed with 4% paraformaldehyde in PBS for 20 min at RT and washed with PBS. The inserts were then incubated with CF-488A phalloidin (1:400) (Biotum) for 30 min and washed with PBS. To analyze the distribution of the ZO-1 protein on the cell monolayers, the inserts were incubated in PBS with 0.25% Triton X-100 for 10 min and blocked with 10% goat serum, 1% BSA, and 100 mM glycine in PBS. The inserts were then incubated overnight at 4°C in a blocking buffer containing anti-ZO-1 polycolonal antibody (2.5 μg/ml) (Invitrogen). After washing, the inserts were incubated in PBS with 5 μM of DRAQ5 to stain the nuclei (Biostatus). The filters were carefully removed from the insert and mounted with a mounting medium (Ibidi). Images were acquired with a Leica SP5 Inverted Confocal and processed and analyzed using ImageJ software.

### Gene expression analysis and protein quantification

Real-time qRT-PCR analysis was performed with Taqman probes after cDNA preparation from cell lysate using a Cell-to-CT kit (Thermo Fisher Scientific) according to the manufacturer’s instructions. Results are expressed as fold increase as compared to controls.

Protein was quantified in the EPs, RBC, and iRBC preparations using the Bradford method.

### Statistical analysis

Data were expressed as mean ± standard deviation (SD). Statistical analysis was carried out by one-way or two-way analysis of variance (ANOVA) as indicated in the figure legends. All statistical tests were performed using GraphPad Prism 6 Software.

## Results

### An in vitro brain endothelial cell barrier system using primary cell cultures

**Fig. 1:**
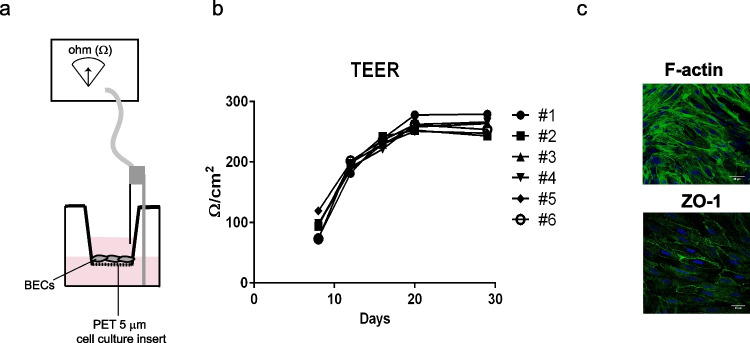
Establishment of a brain endothelial cell barrier in vitro. **a** Schematic diagram of the experimental setup consisting of brain endothelial cells (BECs) isolated from mouse brains and cultured on cell culture inserts with a polyethylene terephthalate (PET) membrane of 5 μm porous to form an endothelial cell barrier. The formation of this endothelial barrier was monitored by transendothelial electrical resistance (TEER) measurements with a voltmeter. **b** Barrier formation in six independent wells (#1–6) showed similar kinetics and was established by day 20 after cell seeding. **c** Immunofluorescence analysis of endothelial cells showing F-actin or ZO-1 staining (green). Nuclei were stained with DRAQ5 (blue)

We aimed at establishing a reproducible and straightforward experimental setup that would mimic the brain endothelial cell barrier and allow studying the impact of *Plasmodium* blood stage factors on brain endothelial cell function. Brain vessels isolated from adult mice were digested with collagenase and directly plated on 24-well culture inserts coated with matrigel. After 6–8 days of culture, transendothelial electrical resistance (TEER) in the inserts was monitored (Fig. 1a). The resistance of the cell culture raised steadily from day 8, until day 20 when it stabilized around 250 Ω/cm^2^ for the next 10 days (Fig. 1b). This value is within the range of 100–300 Ω/cm^2^ previously obtained with the monoculture of brain endothelial cells (Helms et al. 2016). At this stage, immunofluorescence staining for F-actin using phalloidin revealed cells with a spindle-shaped morphology while tight junction protein ZO-1 staining with focal membrane distribution suggested the presence of assembled intercellular junctions (Fig. 1c). Overall, this cell culture system produced a reproducible brain endothelial barrier phenotype characterized by junctional tightness with high and stable TEER levels and focalization of ZO-1.

### iRBCs but not RBCs compromise the brain endothelial cell barrier function

To study the effect of *P. berghei*-iRBCs on the brain endothelial cell barrier, we added synchronized *P. berghei*-iRBCs (1–5×10^6^) to the inserts with established cell monolayers. As a control, we added non-infected RBCs or just medium. We measured TEER before (*t*=0h) and after adding several stimuli (*t*=2, 4, 6, and 24h). After 24 h, we tested the monolayers for permeability to Lucifer yellow (LY) (Fig. 2a).

**Figure:**
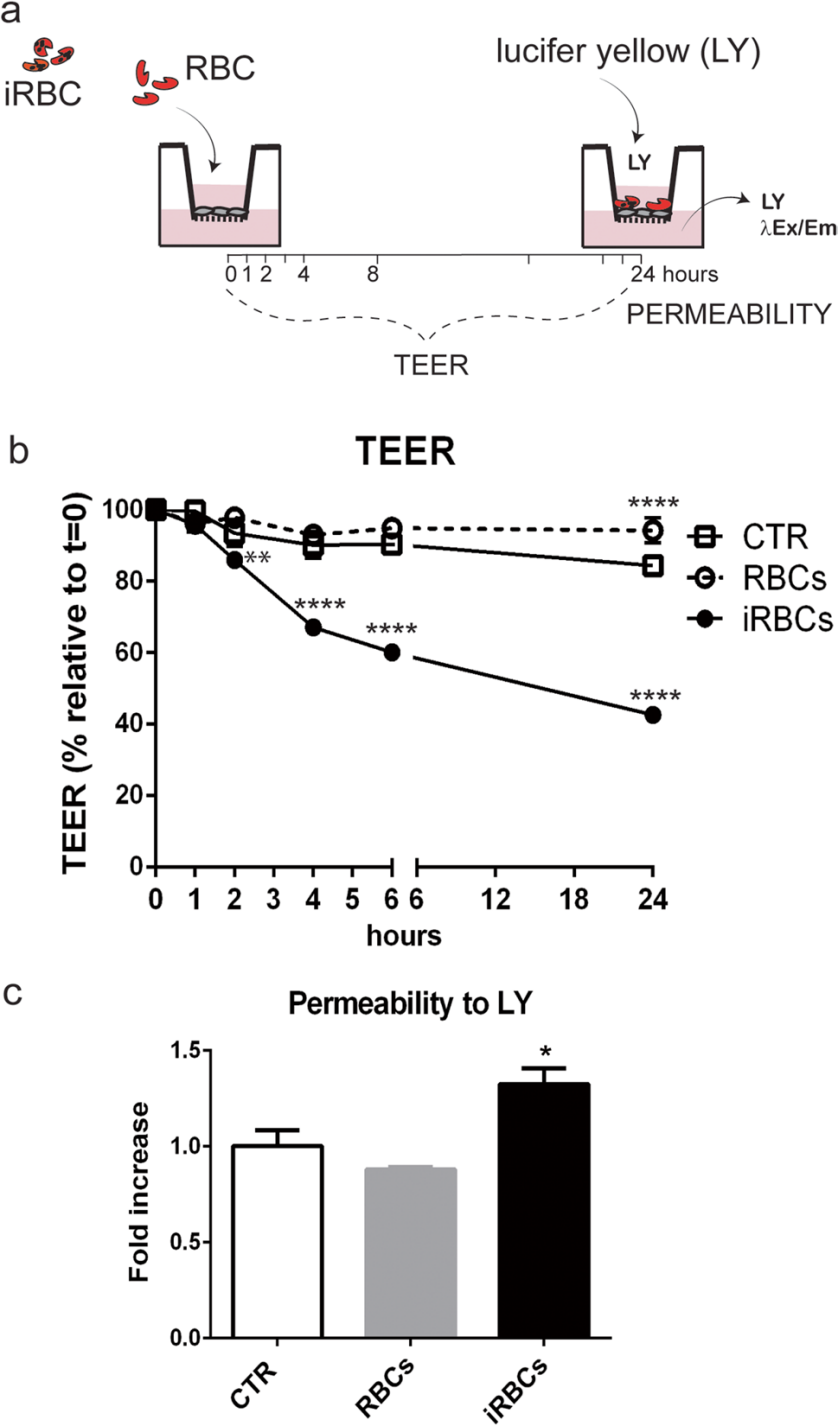
Fig. 2 iRBCs compromise the brain endothelial cell function. a Transendothelial electrical resistance (TEER) was measured before (t=0) and after adding non-infected red blood cells (RBCs), infected red blood cells (iRBCs), or culture medium (CTR) to cultures with established endothelial cell barrier. The permeability to Lucifer yellow (LY) was assessed after 24 h of incubation. b TEER was measured 2, 4, 6, and 24 h after incubation with non-infected RBC, iRBCs, or culture medium (mean±s.d., *n*=3; two-way ANOVA, **P<0.01; **** *P*<0.0001). c Diffusion across the barrier was assessed by detecting LY in the lower chamber 2 h after adding it to the upper chamber of cultures exposed to RBCs, iRBCs, or CTRL for 24 h (mean±s.d., *n*=3; one-way ANOVA, *P<0.05). Data are representative of three independent experiments

After 2 h of incubation with iRBCs, TEER was significantly reduced (~15%) in the endothelial cell cultures (Fig. 2b). The electric resistance progressively decreased during the following hours reaching approximately 40% of the values measured in unexposed cells after 4 h of incubation, an indication that iRBCs can induce rapid loss of brain endothelial barrier function. To test for barrier permeability, we used the low molecular weight marker LY at 24 h of iRBC exposure, when the reduction of TEER exceeded 50%. Dye diffusion to the lower chamber after 2 h was significantly higher in endothelial cells exposed to iRBCs than in non-exposed cells, which indicates increased intercellular permeability and loss of brain endothelial barrier function (Fig. 2c).

### iRBCs induce alterations of endothelial tight junctions and upregulate mRNA levels of pro-inflammatory Icam1

**Fig. 3:**
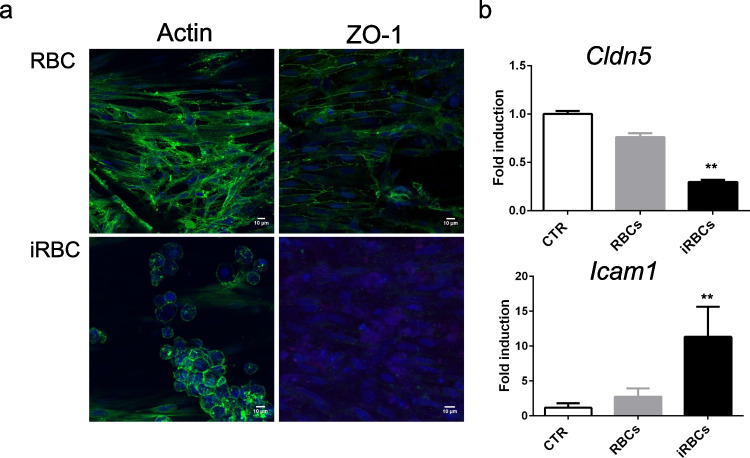
iRBCs alter cell morphology and expression of tight junction components and adhesion molecules genes in brain endothelial cells. **a** As described in Fig. 1c (F-actin and ZO-1), immunofluorescence staining revealed alterations in cell morphology in the distribution of the tight junction protein ZO-1 in cultures incubated with infected red blood cells (iRBCs) for 24 h. **b** Relative quantification of *Cldn5* and *Icam1* mRNA in cultures of endothelial cells barrier exposed to non-infected (RBCs), iRBCs, or culture medium (CTRL) for 24 h (mean±s.d., *n*=3; one-way ANOVA, **P<0.01)

Loss of the brain endothelial barrier induced by iRBCs was associated with alterations in endothelial cell morphology. After 24 h of exposure to iRBCs, endothelial cells had lost their typical elongated morphology and were clustered together as shown by F-actin staining (Fig. 3a). In addition, staining for ZO-1 was decreased suggesting disruption of intercellular tight junctions (Fig. 3a). This experimental setup also allows collection of endothelial cells for molecular analysis. Gene expression analysis of endothelial cells exposed to iRBCs revealed decreased mRNA expression of the brain endothelial tight junction protein claudin-5 while *Icam1* mRNA was upregulated (Fig. 3b). These results indicate that iRBC exposure activates endothelial cells and compromises tight junction maintenance.

### iRBC-derived EPs are sufficient to induce brain endothelial cell barrier dysfunction

**Fig. 4:**
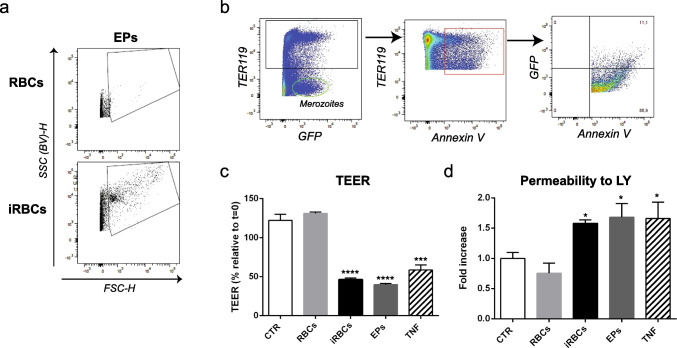
Blood stage-derived EPs decrease TEER values. **a** Extracellular particle (EP) preparations from the supernatant of infected red blood cell (iRBC) cultures were detected by flow cytometry using side scatter height from the blue-violet laser, SSC (BV)-H, and forward scatter height, FSC-H. **b** The EP preparation contained GFP^+^ particles corresponding to *P. berghei*-GFP merozoites that do not stain for TER119 as well as TER119^+/^Annexin V^+^ particles that contain Pba-GFP-derived products. **c** The effect of EPs in the endothelial barrier was evaluated by transendothelial electrical resistance (TEER) measurements and **d** permeability assay to Lucifer yellow (LY) after 24 h of incubation with red blood cells (RBCs) (5×10^6^), iRBCs (5×10^6^, 111 μg/ml), EPs (183 μg/ml), or TNF (10 ng/ml) (mean±s.d., *n*=3; one-way ANOVA, *P<0.05 and ****P<0.001). Data are representative of three independent experiments

Human and experimental cerebral malaria induces an increase in plasma levels of cell-derived membrane vesicles (0.1–1 μm in diameter), including extracellular vesicles derived from iRBCs (Pankoui Mfonkeu et al. 2010; El-Assaad et al. 2014). Rather than just a disease epiphenomenon, these vesicles may carry factors (lipids, proteins, nucleic acids) with biological activity that contribute to CM pathogenesis and in particular to brain vascular alterations. We have collected the supernatant of synchronized cultures of iRBCs, which is enriched in EPs when compared with the supernatant of non-infected RBCs as determined by flow cytometry analysis (Fig. 4a). This EP preparation contained free merozoites, defined in flow cytometry analysis as GFP^+^ particles, derived from the Pba-GFP parasite, and negative for TER119, a membrane protein expressed by RBCs (Fig. 4b). In addition, we also identified a population of EPs stained for Annexin V, a marker of extracellular vesicles (El-Assaad et al. 2014). A fraction of this population exhibit a GFP signal probably derived from the Pba-GFP parasite (Fig. 4b). We found that this heterogeneous mixture induced a decrease in TEER comparable to the one observed upon 24 h of incubation with iRBCs (Fig. 4c). These effects were similar to the alterations in TEER induced by tumor necrosis factor (TNF), a cytokine that elicits endothelial cell inflammatory responses. We further confirmed previous data showing that TNF disrupt the endothelial cell barrier in vitro (De Vries et al. 1996). In agreement with the loss of TEER, the permeability to LY increased in the stimulated cells (Fig. 4d). Thus, usage of our experimental setup allows kinetic analysis and quantitative comparisons of factors impinging endothelial cell barrier disturbances, being an important tool to uncover mechanisms of BBB disruption in CM.

## Discussion

The mechanisms that lead to BBB disruption in CM involve both host inflammatory mediators and parasite-derived factors. iRBCs, especially those that contain mature forms of the parasite, may activate mechanisms that contribute to the loss of endothelial barrier function. Here, we established a robust in vitro experimental setup to study direct (cell to cell) and indirect (through the release of bioactive factors) iRBC-endothelium interactions. This model avoids the exposure of animals to CM symptoms while enabling the study of several parameters related to BBB disruption during CM such as loss of barrier function, endothelial cell morphology alterations, and expression of activation markers.

Experimental mouse models of CM evidence the relation between disease development and the presence of iRBCs in the brain, which are trapped in post-capillary venules (Ball et al. 2013). Whether *P. berghei*-iRBCs bind to specific receptors on the mouse brain endothelium remains unclear. Nevertheless, iRBCs may regulate endothelial cell barrier function through direct contact or by releasing soluble and insoluble factors during parasite maturation. Studies with human endothelial cell lines (HBEC-5i and hCMEC/D3) and primary human brain microvascular endothelial cells revealed that *Pf*-infected RBCs adhere to these cells and regulate tight junction proteins, pro-inflammatory factors, TEER, and permeability to solutes (Polimeni and Prato 2014). However, the cell lines were derived from brain microvessels after immortalization with the viral SV40 large T antigen known to affect innate immune response pathways (Smith et al. 2013). Also, immortalized endothelial cells reach lower levels of TEER compared to primary cells (Omidi et al. 2003). On the other hand, there is also a considerable variation between human primary endothelial human cell batches and controversy around the endothelial cell properties of human induced pluripotent stem cell-derived brain endothelial cells (Lu et al. 2021). We used an optimized commercial medium for endothelial cells to produce an in vitro brain endothelial barrier that potentially overcomes these pitfalls and offers a reproducible system to unveil molecular mechanisms involved in tight junction disruption underlying BBB leakage in CM. To illustrate this, we showed here that similarly to iRBCs, EPs derived from iRBCs significantly reduce the TEER of the endothelial cell barrier.

Induction of endothelial cell death by the interaction with iRBCs may cause impairment of the endothelial cell barriers during *Plasmodium* infection as suggested by studies with both pulmonary and brain endothelial cells. The results depended on the type of endothelial cell, culture conditions, and *Plasmodium* parasites (N’Dilimabaka et al. 2014). Other studies showed that iRBCs induce lung endothelial cell apoptosis through caspase 3, 8, and 9 activation with increasing cellular permeability (Pino et al. 2003; Sercundes et al. 2022). Interestingly, apoptotic brain endothelial cells were not associated with loss of BBB integrity in mice with cerebral malaria (Shaw et al. 2015). In our experiment setup, nuclei staining of brain endothelial cells incubated with iRBC did not show typical pyknosis of cells undergoing necrosis or apoptosis. In contrast, F-actin staining indicates alterations in the endothelial cell cytoskeleton with loss of the characteristic spindle-shaped morphology and an increase in the intercellular space. Future work may reveal if specific parasite-derived factors induce caspase activation or affect actin polymerization and tight junction proteins leading to endothelial barrier disruption and a decrease in TEER. Concomitantly, using endothelial cells from mice deficient in specific innate immune recognition receptors in this experimental setup will facilitate the identification of new mechanisms involved in endothelial activation and barrier dysfunction relevant for CM pathogenesis. Importantly, the model also allows adding to the upper chamber (mimicking the microvessel luminal space) other inflammatory stimuli or other cells that interact with endothelial cells during CM development such as TNF. In addition, this model can be used in preclinical studies for in vitro screening of drugs with potential effects on the endothelial barrier, prior to in vivo experimentation in mouse models of diseases affecting BBB. Such strategy could both increase the likelihood of selecting a drug that would effectively target the BBB and significantly reduce the number of animals used in experimentation.
